# Remarks on Making Gold Plate and Solder

**Published:** 1852-10

**Authors:** B. Wood

**Affiliations:** Dentist


*Remarks upon making Gold Plate and Solder.* By B. Wood, M. D., Dentist.

Messrs Editors:—Having recently made some experiments in the way of preparing gold plate and solder, I take the liberty of submitting the result, with the hope that it may elicit further information from some of your readers who may be more conversant with the working of metals.

*Gold Plate.*—The ordinary alloy of eighteen to twenty, or even twenty-one carats, for gold plate, is liable to objection.—In some mouths, it will tarnish, and even corrode. A plate not liable to this, may be made by alloying pure gold with a small proportion of platinum; and for the past few years, this expedient has been resorted to by dentists. Still, the plate thus made is objectionable. If enough platinum is used to give the necessary elasticity, &c., it impairs the color, as well as the susceptibility of receiving a good polish. Besides the peculiar gray and almost dirty hue is apt to suggest the suspicion that the plate is base. But a few days ago, I was called upon to testify, for a worthy professional brother, to the fineness of the gold used in an artificial set of teeth, the plate of which was alloyed in this way. It appears the patient had exhibited the work to a quack dentist, who, doubtless, because the gold did not, in point of color, compare well withhis six teen or eighteen carat (alloyed with copper and silver) succeeded in making the impression that it was very impure.

The object of my experiment was to obtain a plate which, while as fine as possible, should possess the requisite hardness and elasticity, retain the natural gold color, and be susceptible of the desired polish. The following proportions I find to answer these ends better than any other that I have tried:

By weight: 45 to 50 parts of pure gold.

" " 2 " " silver.

" " $\frac{3}{4}$ " " copper.

" " $\frac{1}{4}$ " " platinum.

I first prepare the alloy as follows: platinum 1 part; copper, 3 parts; silver 8 parts, (or from 6 to 8 parts.) This forms a very pretty alloy, of a bright silver color, with a yellow tinge. It is quite hard, and of a firm, close structure: malleable, and bears a fine polish. With this, I alloy the gold, to from $21\frac{1}{2}$ to $22\frac{1}{2}$ carats, according to the fineness required, (by adding $2\frac{1}{2}$ or $1\frac{1}{2}$ parts as the case may be, to $21\frac{1}{2}$ or $22\frac{1}{2}$ of pure gold).

It may not be known to all of your readers that platinum can be made to unite directly with gold, as also with silver and copper, by the heat of the common blow-pipe. Many dentists are in the habit of filing platinum, in order to melt it with gold, and some are under the belief that, even then, it does not really fuse, but rather *mixes* with the latter, which has frequently led them to throw away their chipings from the rivits of artificial teeth, lest the presence of such should spoil their plate. Although always satisfied in regard to the actual fusion of platinum, with gold, yet I confess, laboring under the impression that, at the ordinary heat, this took place only at the surfaces of contact, and that hence it was necessary the platinum be in a state of minute division, as in the form of filings, in which I used it. Nor was this error corrected, until after trying some quite coarse filings with gold, when, finding this to fuse under the blow-pipe, I tried next rivet chippings, and then larger masses of platinum with the same success.

The platinum *amalgamates*, as it were, with the fused metal, in the same way that gold combines with melted tin.

All that is necessary, is to put your scraps to be fused into a depression made in a piece of charcoal, then apply an intense heat by means of the blow-pipe. In alloying platinum with copper, in this way, it is well to cover in part with another coal, that the copper may not oxydize too soon. In this manner, every one can make his own allow, and then, if he chooses, send the proper proportions to the goldsmith for melting and rolling.

I have frequently made inquiries of dentist, (whom I supposed likely to be informed on the subject), in regard to the mode and proportion of mixing platinum with gold, but without obtaining any thing satisfactory; it may have been from a want of the knowledge sought after, or to a disposition to keep it a "*secret.*" In either case, the above hints may not be without value to some in the profession. At any rate, there are many things which, though they should not prove to be *new*, it is well to bring into notice anew, so that they be not forgotten. A case in point, is the mode of "welding' platina, recently communicated to the profession by Dr. J Allen, of Cincinnati, but which was performed in the time of M. Delabarre. "In order to accomplish it, he placed a very thin gold plate, twenty carats fine, between the two leaves of platina, fastened them by two rivets, and soldered them by the fusion of the intermediate leaf."* This is upon the same principle of that published in the *New York Dental Recorder* for February, viz: by *soldering* through the means of gold. And yet, Dr. Allen none the less deserves our thanks for suggesting its application, in uniting together platina fragments, otherwise useless for the purposes in which this metal, as a mass, comes into requisition.

*Gold Solder as fine as the Plate.*—From various experiments, I find the following answers best in point of color, &c., for the above plate: pure *gold*, 22 parts; *tin*, 1 part; *zinc*,

**Desirabodess Scienc eand Art of the Dentist;* Dental Library Ed. page 430

1 part. This, by annealing, is sufficiently malleable for solder; melts easily enough, and flows well. It will be seen that *it is twenty-two carats fine.*

Judging from a series of experiments in making solder with tin, zinc, brass, copper, &c. I am led to believe that copper adds nothing to the fusibility of the alloys, nor assists in making it flow better. It would seem to be the combination of *zinc* that gives the *flow.*

The advantages of this solder are, 1st, its fineness; 2d, its being of the same standard as the plate used, thereby obviating the galvanic action resulting from a difference in fineness of plate and solder; 3d, it is without the copperish taste so frequently experienced from solder alloyed with copper; and 4th, it has not the hardness which this metal communicates, but on the other hand, can be easily filed or cut, and leveled off, smoothed, and polished as the plate itself.

I first make an alloy of four parts gold (pure); one tin, and one zinc. This forms a grayish, white, and very brittle mass. Although sixteen carats fine—or as fine as *jewellers' gold,* which some "dentists" use for plate!—it crumbles under the hammer, and melts very readily. Of this, take one part to three of gold, and you have the above.—*Phila. News Letter.*

